# Evaluation of Proximate, Phytochemical, and Heavy Metal Content in Black Cumin and Fenugreek Cultivated in Gamo Zone, Ethiopia

**DOI:** 10.1155/2023/3404674

**Published:** 2023-01-13

**Authors:** Alemu Mekonnen Tura, Markos Dele Debisa, Etalema Desta Tulu, Birhanu Zeleke Tilinti

**Affiliations:** Arba Minch University, College of Natural and Computational Sciences, Department of Chemistry, Arba Minch, P.O. Box 21, Ethiopia

## Abstract

Spices have been recognized to have medicinal properties. Among several spices and medicinal plants, black cumin and fenugreek are very common. Geographical, climatic, and cultivating soil type can change the chemical composition of these spices. The objective of this work is to determine proximate, phytochemical, and heavy metal content in black cumin and fenugreek cultivated in the Gamo zone, Ethiopia. The proximate composition (moisture, ash, fiber, fat, protein carbohydrate, and gross energy) was determined; their content (%) in black cumin is 6.98 ± 0.12, 5.02 ± 2.9, 6.90 ± 0.21, 32.1 ± 0.68, 20.9 ± 0.09, 34.1 ± 0.73, and 498 ± 4.4, respectively, and 6.30 ± 0.35, 4.27 ± 0.17, 9.36 ± 0.25, 12.8 ± 0.41, 30.8 ± 0.09, 46.1 ± 0.52, and 422 ± 1.6, respectively, in fenugreek. The total means of phenolic flavonoids and alkaloids in black cumin are 193 ± 5.3 mg GAE/100 g, 87.6 ± 4.3 mg QE/100 g, and 69.4 ± 4.7 mg AE/100 g, respectively, and 382 ± 11 mg GAE/100 g, 123 ± 3.6 mg QE/100 g, and 37.6 ± 2.2 mg AE/100 g, respectively, in fenugreek. In black cumin and fenugreek collected from Kamba, Daramalo, and Dita woredas, nine heavy metals were determined by using FAAS. The total mean concentrations in mg/kg for detected heavy metals (Fe, Zn, Cu, Mn, and Co) in black cumin are 78.5 ± 5.9, 27.3 ± 1.5, 3.06 ± 0.26, 8.47 ± 0.22, and 10.1 ± 0.37, respectively, and Cr, Ni, Pb, and Cd were not detected in black cumin. Similarly, the concentrations of Fe, Zn, Cu, Mn, Co, and Ni in fenugreek are 168 ± 5.2, 14.8 ± 0.20, 4.76 ± 0.1, 13.7 ± 0.40, 3.66 ± 0.21, and 2.12 ± 0.03, respectively, and Cr, Pb, and Cd were not detected in fenugreek. All the results of determined parameters were compared to previous studies, and the values were in agreement with slight variations. Therefore, black cumin and fenugreek cultivated in the Gamo zone were free from heavy metal toxicity.

## 1. Introduction

The World Health Organization (WHO) recently estimated that 80% of the world's population relies in some way on medicinal plants for their basic healthcare [1]. Medicinal plants naturally create and accumulate secondary metabolites like phenols, alkaloids, steroids, terpenoids, flavonoids, saponins, glycosides, and tannins that fight disease [2]. Spices have been shown to have therapeutic effects because of their antioxidant, antimicrobial, antidiabetic, anti-inflammatory, and chemoprotective properties [3]. Due to their accessibility, effectiveness, and cost, spices are frequently utilized as medicines in Ethiopia [4]. Two spices and healing plants, black cumin and fenugreek, have a long history of usage in traditional medicines all around the world [5]. Both culinary and therapeutic uses have been made of the seed of black cumin, *Nigella sativa*, a perennial flowering plant in the Ranunculaceae family. Numerous other names, such as black cumin (English), black-caraway (USA), habba-tusawda (Arabic), tikur azimud (Amharic), and karetta shuquwa (Wolaita and Gamo), are also widely used to refer to black cumin [6]. Because of their dark hue and bitter flavor, black cumin seeds are used as flavoring in curries, sweets, cheeses, and soups. It can also be added to bread, tea, coffee, and tinned products. Back cumin has a long history of use as a medicinal herb in a number of traditional herbal systems to treat a wide range of ailments, including diabetes, paralysis, airway disorders, inflammation, infections, hypertension, and problems relating to the digestive tract, as well as pain conditions like chronic headaches and back pain [7].

The annual flowering plant known as fenugreek, or *Trigonella foenum-graecum*, belongs to the Fabaceae family. Fenugreek is a native of the Eastern Mediterranean region and is widely cultivated in Central Asia, Pakistan, India, and China. Fenugreek is known by many different names in different parts of Ethiopia, including Sunqoo (Afan Oromo), Abake (Tigrigna), Abeshe (Guraginga), Abish (Amharic and Kore), and Shuquwa (Wolaita and Gamo). Around the world, fenugreek leaves and seeds are used for a variety of things, including food preparation (like making cheese soup and bread), dyeing, controlling insects in grain storage facilities, preventing blood poisoning from wounds, and medicinal purposes (like being antidiabetic, anticancer, and antimicrobial; lowering blood sugar and cholesterol levels; and preventing blood poisoning from wounds) [8].

Spices are functional foods, which are foods that, in addition to providing basic nutritional needs, have been found to positively affect a number of important body systems. Black cumin and fenugreek are used by more than 85% of Ethiopians to season food, improve the flavor of beverages like coffee and tea, and treat all of their medical conditions [9]. Spices may operate as a channel for the transfer of toxins and heavy metals from the environment to individuals through the food chain because they are derived from plants [10, 11]. Consuming medicinal herbs or spices that have accumulated high traces of hazardous metal pollution over an extended period of time may cause these compounds to bioaccumulate in the body's organs, which can result in a number of health problems [12]. Understanding the amounts of phytochemicals and heavy metals in these spices is essential given the widespread intake of spices. The results of this type of research may therefore serve as a guide or informational source for those who eat these spices, providing them with vital information regarding the safety of these substances in terms of their effects on nutrition and health [13, 14].

Black cumin and fenugreek grown in the Gamo zone must therefore be assessed for the level of proximate values, phytochemicals, and necessary and hazardous heavy metals in order to estimate (limit) the daily intake. There have been numerous studies on the topic of proximate, phytochemical, and metal determination in black cumin and fenugreek grown in various countries around the world, but fewer studies have been conducted in Ethiopia. However, there have been no studies on black cumin and fenugreek grown in the Gamo zone for their proximate, phytochemical, and heavy (essential and toxic) metal levels. Therefore, the present study is intended to fulfill these research gaps. The aim of the present study was to evaluate the proximate, phytochemical, and heavy metal in black cumin and fenugreek cultivated in Gamo zone, Ethiopia.

## 2. Materials and Methods

### 2.1. Sample Collection and Preparation

Three separate districts in the Gamo zone were used to gather the samples (i.e., Kamba, Daramalo, and Dita). Farmers in each chosen district were asked to donate 500 grams each of black cumin and fenugreek, which were then taken to the chemistry lab at Arba Minch University. The samples were cleaned with tap water, dried in the sun for two days, and then ground into a fine powder using a mortar and pestle. They were then placed in clean polyethylene bags and kept in a clean, dry, and cool location until their next usage.

### 2.2. Proximate Composition Determination

The proximate compositional analysis of black cumin and fenugreek seeds, including percentages of fat, fiber, protein, carbohydrate, moisture, ash, and energy, was determined by using AOAC [15–18] guidelines.

### 2.3. Determination of Phytochemicals in Black Cumin and Fenugreek

#### 2.3.1. Sample Preparation for Phytochemical Determination

Because polar phytochemicals like phenols are extracted within polar solvents and nonpolar phytochemicals like alkaloids are extracted more readily, methanol (a polar solvent) and petroleum ether (a nonpolar solvent) were utilized separately for extraction purposes. 25 grams of each sample was put into a 400 mL extraction flask, which was then filled with 200 mL of methanol and petroleum ether. The mixtures were then agitated at room temperature for three days before being filtered through Whatman paper no. 1 and evaporated using a rotary evaporator. For additional analyses, dried extract was stored in a refrigerator at 4°C. Finally, distilled water containing 1 mg/mL (*w*/*v*) of each extract was added for the phytochemical test. The crude extracts of black cumin and fenugreek samples were tested for the presence of selected major bioactive compounds like phenolic compounds, alkaloids, flavonoids, saponins, tannins, and terpenoids by using following standard methods [19–22].

### 2.4. Quantitative Determination of Some Phytochemicals

#### 2.4.1. Determination of Total Phenolic Content (TPC)

The Folin-Ciocalteu method, as described in [16], was used to quantify the total phenol content of the methanol extract of the samples of cumin and fenugreek. Gallic acid was used as the reference phenolic component while drawing the standard calibration curve. The Folin-Ciocalteu reagent was newly made and used at a ratio of 1 : 10 with distilled water [23].

*(1) Preparation of Standard Solution*. 10 mg of standard gallic acid was accurately weighed and dissolved in 100 mL distilled water in a volumetric flask to obtain 100 ppm, and then, 1.5, 3.75, 6.25, 8.75, and 11.25 mL of aliquots were pipetted out into 25 mL volumetric flasks to prepare 5 ppm, 15 ppm, 25 ppm, 35 ppm, and 45 ppm of atropine solution, respectively. Then, 10 mL of distilled water and 1.5 mL of Folin-Ciocalteu reagent were added to each of the above volumetric flasks. After 5 min, 4 mL of 0.7 M sodium carbonate was added, and then, distilled water was added to increase the volume up to 25 mL. A dilute methanol extract (0.5 mL of 1 : 10 mg mL) was mixed with the Folin-Ciocalteu reagent (5 mL, 1 : 10 diluted with distilled water) and aqueous Na_2_CO_3_ (4 mL, 1 M). The resultant mixes were allowed to stand for 15 min before the colorimetric technique at 765 nm was used to determine the total phenol content. The total phenol values are reported in terms of gallic acid equivalent (mg GAE/100 g of dry mass), and the total phenol levels of the methanol extract were determined using a standard calibration curve. The total phenol content of each sample was determined in triplicate and quantified using the gallic acid standard curve. The results were represented as milligrams of gallic acid equivalents (GAE) per 100 grams of extracts [23].

(1) $$\begin{array}{c} \text{Total phenol content}\left(\text{mg GAE}/100\text{ g}\right)=\frac{\text{Concentration}\left(\text{mg}/\text{L}\right)\times \text{Volume of flask }\left(\text{L}\right)\times \text{Df}}{\text{Sample mass }\left(\text{g}\right)}, \end{array}$$

where Df is the dilution factor.

#### 2.4.2. Determination of Total Flavonoid Content (TFC)

*(1) Preparation of Standard Solution*. To create an intermediate standard solution containing 100 ppm of quercetin, 10 milligrams of the antioxidant was precisely measured and diluted in 100 mL of distilled water. Then, five series of quercetin working standards were created using the dilution equation (*C*_1_*V*_1_ = *C*_2_*V*_2_); 2.5, 5, 7.5, 10, and 12.5 mL were pipetted into a 25 mL volumetric flask to provide standard solutions of 10, 20, 30, 40, and 50 ppm of quercetin, respectively. Plotting an absorbance versus concentration calibration curve was done. Using quercetin as a reference flavonoid component, the total flavonoids in petroleum ether extracts of black cumin and fenugreek samples were calculated [24]. Sample extracts of 0.5 mL (1 mg/mL) were separately mixed with 0.15 mL of 10% aluminum chloride and 0.5 mL of 1 M sodium acetate, and the final volume of mixture was made 2.5 mL with distilled water mixed thoroughly and incubated for 30 min at room temperature. The absorbance of the mixture was measured at 415 nm against a blank solution with a UV-visible spectrophotometer. The results were expressed as milligram of quercetin equivalent (QE) per 100 g of dry matter.

(2) $$\begin{array}{c} \text{Total flavonoid content }\left(\text{mg QE}/100\text{ g}\right)=\frac{\text{Concentration}\left(\text{mg}/\text{L}\right)\times \text{Volume of flask }\left(\text{L}\right)\times \text{Df}}{\text{Sample mass }\left(\text{g}\right)}. \end{array}$$

#### 2.4.3. Determination of Total Alkaloid Content (TAC)

*(1) Preparation of Standard Solution*. 10 mg of atropine was measured and diluted in 100 mL of distilled water to make intermediate standards (100 ppm). Next, atropine intermediate standard solutions of 1.25 mL, 2.5 mL, 3.25 mL, 5 mL, and 6.25 mL were pipetted into 25 mL volumetric flasks to make working standard solutions of 5, 10, 15, 20, and 25 ppm, respectively. Finally, 5 mL of phosphate buffer (pH 4.7) was prepared. Then, a calibration graph was created after measuring the absorbance at 470 nm of wavelength. The total alkaloids in methanol extracts of black cumin and fenugreek were calculated using the atropine standard calibration curve. 25 mL of the volumetric flask was filled with samples containing 1 mL (1 mg/mL) of 2 M HCl, which was then dissolved and filtered to determine the total alkaloid content. By adding 0.1 N NaOH, the pH of the extract was brought to neutral. 5 mL of bromocresol green and 5 mL of phosphate buffer were added to 1 mL of this solution, and the mixture was thoroughly mixed. The mixture was subjected to further extraction using 5 mL of chloroform, transferred to a 25 mL volumetric flask, and brought to the desired level with the same solvent. At 470 nm, the complex's absorbance was measured. Milligrams of atropine equivalent (AE) per 100 g of dry matter were used to express the results [25].

(3) $$\begin{array}{c} \text{Total alkaloid content }\left(\text{mg AE}/100\text{ g}\right)=\frac{\text{Concentration}\left(\text{mg}/\text{L}\right)\times \text{Volume of flask }\left(\text{L}\right)\times \text{Df}}{\text{Sample mass }\left(\text{g}\right)}. \end{array}$$

### 2.5. Determination of Selected Heavy Metals

#### 2.5.1. Sample Digestion

For determination of heavy metals in black cumin and fenugreek, samples collected from three different woredas of the Gamo zone (Kamba, Daramalo, and Dita) were washed with tape water, dried in the sun for two days, ground with a mortar and pestle (homogenized) to fine powder, sieved in a 0.5 mm mesh, digested in acids, and analyzed individually without mixing. The sample digestion procedure was used using [26]; 0.5 g of dry material prepared in a beaker was introduced to 6 mL of concentrated nitric acid (69% HNO_3_) and 4 mL of concentrated perchloric acid (70% HClO_4_). After 30 minutes of open acid digestion, the mixture was heated for 40 minutes on a hot plate by gradually raising the temperature from 100 to 1500°C, then cooled down. Next, 2 mL of HClO_4_ and 1 mL of hydrogen peroxide (30% H_2_O_2_) were added, and the mixture was reheated to facilitate complete oxidation of organic substances until the reddish-brown nitrous oxide fume disappeared, leaving a colorless digestion solution. Finally, the mixture was cooled. After acid digestion, a colorless solution was obtained and filtered into a 50 mL volumetric flask by using filter paper and then diluted to the mark of the flask with distilled water. A blank solution was prepared in the same way as the sample preparation except for the addition of the sample.

#### 2.5.2. Preparation of Standard Solutions of Heavy Metals

The intermediate standard solution of each metal was prepared from a stock standard solution (1000 mg/L) by taking 10 mL in a 100 mL volumetric flask. Five series of working standard solutions of each heavy metal were prepared from the intermediated standard solutions (100 mg/L) by using the dilution equation (*C*_1_*V*_1_ = *C*_2_*V*_2_) as indicated in Table 1.

### 2.6. Statistical Analysis

The variance analysis, a highly effective statistical method, was used to assess the differences between the means of three sets of measurements (ANOVA). Using software from the Statistical Package for Social Sciences, the mean concentration of experimental data from the analysis of black cumin and fenugreek was compared using one-way ANOVA at a 95% confidence level (SPSS).

## 3. Results and Discussion

### 3.1. Proximate Composition of Black Cumin and Fenugreek

The proximate compositions of black cumin (*Nigella sativa*) and fenugreek (*Trigonella foenum-graecum*) seed collected from three different woredas of the Gamo zone were determined, and the results were reported as %mean ± %standard deviation as shown in Table 2.

#### 3.1.1. Moisture Content

The average moisture content in percent of black cumin collected from Kamba, Daramalo, and Dita was 6.40 ± 0.10%, 6.69 ± 0.0643%, and 6.92 ± 0.04%, respectively. Black cumin from Dita has the highest and the lowest moisture content, and the average of the three cultivars was 6.69 ± 0.22%. The mean moisture content of three cultivars of black cumin was not significantly different at the 0.05 level (*p* = 0.53058, which is *p* ≥ 0.05). The average moisture content in back cumin of this study (6.69 ± 0.22) was lower than the literature mean value (7.12) studied in Bangladesh [27] and higher than that (4.2 ± 0.3) reported by [28]. The moisture of the fenugreek sample collected from Kamba, Daramalo, and Dita was 6.08 ± 0.05%, 5.90 ± 0.07, and 5.99 ± 0.14, respectively. Fenugreek from Kamba has the highest moisture content, and fenugreek from Dita has the lowest moisture content with an average moisture content of three cultivars of6.29 ± 0.34%. At the 0.05 level, the means are not significantly different (*p* = 0.6699, which means *p* > 0.05). When the moisture content values of black cumin and fenugreek were compared, at the 0.05 level, the means were significantly different (*p* = 0.01239, which implies *p* < 0.05). The moisture content of fenugreek (6.299 ± 0.349) in this study was smaller than the literature mean value (7.54) reported by [29] studied in Ethiopia and lower than the literature mean value (8.1 ± 0.3) reported by [30] studied in Ethiopia. This may be due to differences of concentration of minerals in cultivation area soil and climatic condition.

#### 3.1.2. Ash Content

The ash content is a measure/reflection of the mineral content present in the food material. The ash content of black cumin collected from Kamba, Daramalo, and Dita woredas was 5.23 ± 0.04, 5.05 ± 0.02, and 4.78 ± 0.13, respectively. At the 0.05 level, the means were not significantly different (*p* = 0.92009, which is *p* > 0.05). The average ash content in black cumin (5.017%) of this study was smaller than the literature mean result (7.39) stated by [27] and higher than the literature mean value of ash content (4 ± 0.3) reported by the study in [28] in Ethiopia. The ash content of the three cultivars of black cumin was consistent with that reported for black cumin in Pakistan [31]. The ash content of the fenugreek samples collected from Kamba, Daramalo, and Dita woredas was 4.27 ± 0.04, 4.44 ± 0.06, and 4.104 ± 0.14, respectively. At the 0.05 level, there was no significant difference between mean ash content of three cultivars of fenugreek (*p* = 0.20483, which was *p* > 0.05). The ash content of fenugreek was significantly smaller than that of black cumin. The mean value ash content in fenugreek of this study (4.27 ± 0.17) was greater than the literature mean value of ash content (3.00) reported by [29] studied in Ethiopia and also greater than the literature mean value (2.6 ± 0.2) reported by [30]. Thus, there was a significant difference between the literature mean value and the ash content in this study. This may be due to climatic, geographical, and mineral content differences of the cultivation area.

#### 3.1.3. The Crude Fiber Content

Dietary fiber promotes growth and protects the beneficial intestinal flora. The fiber content of black cumin collected from three woredas of the Gamo zone (Kamba, Daramalo, and Dita) was 6.84 ± 0.28%, 6.80 ± 0.29%, and 7.068 ± 0.083%, respectively, with an average mean fiber content of three cultivars of 6.90 ± 0.21%. The highest fiber content (7.07 ± 0.08) was investigated in the black cumin from Dita, and the lowest fiber content (6.80 ± 0.29) was observed in the black cumin from Daramalo. At the 0.05 level, the means were not significantly different (*p* = 0.9832, which is *p* > 0.05). The average crude fiber content of black cumin (6.902 ± 0.209%) in this research was greater than the literature values (5.1 ± 0.3) reported by [28] and similar to literature values of [30] studied in Pakistan. The crude fibers of fenugreek samples collected from Kamba woreda, Daramalo woreda, and Dita woreda were 9.48 ± 0.302%, 9.445 ± 0.34%, and 9.16 ± 0.36%, respectively, with a total mean fiber content in three cultivars of fenugreek of 3.09 ± 0.01%. The highest fiber content (9.48 ± 0.30%) was determined in fenugreek from Kamba, and fenugreek from Dita has the lowest fiber content (9.16 ± 0.36%). At the 0.05 level, the means were not significantly different (*p* = 0.65697, which was *p* > 0.05). The total mean fiber content in fenugreek of this study (9.36 ± 0.25%) was significantly greater than the fiber content of black cumin. The total mean fiber content in fenugreek of this study was greater than the literature mean value of fiber content (7.00%) reported by [31] in Ethiopia and much smaller than the literature value (17.0%) reported by [32]. Thus, this significant difference in fiber content between the literature mean value and this study's mean value was due to the variation in mineral content of the cultivation area. In general, the fiber content in black cumin and fenugreek cultivated in the Gamo zone was similar to that in the study reported by [33].

#### 3.1.4. The Crude Fat Content

As shown in Table 2, the mean percentages of crude fat in black cumin from Kamba, Daramalo, and Dita were 32.1 ± 0.53%, 33.5 ± 0.25%, and 30.8 ± 0.19%, respectively, with an average mean of three cultivars of 32.1 ± 0.68%. The black cumin from Daramalo had the highest fat content and that from Dita had the lowest mean fat as compared to their experimental results, and at the 0.05 level, the means were not significantly different (*p* = 0.88556, which is *p* > 0.05). When the crude fat content of this study was compared with different literature values, the average mean values of the three cultivars of black cumin (32.128%) were smaller than the value of the mean fat content in black cumin (45.4%) reported by [34] studied in Bangladesh, and the crude fat content of this research was also in agreement with the study result (32.74%) reported in Pakistan [30]. The mean percentages of crude fat for fenugreek samples collected from Kamba, Daramalo, and Dita were 13.3 ± 0.92%, 11.5 ± 0.22%, and 13.5 ± 0.27%, respectively. The highest mean value fat content (13.5 ± 0.27%) was observed in fenugreek from Dita, and the lowest mean fat content of11.5 ± 0.22%was determined in fenugreek from Daramalo. At the 0.05 level, the means are not significantly different (*p* = 0.6991, which was *p* > 0.05). The mean value fat content of three cultivars of fenugreek in this study (12.8 ± 0.41%) was greater than the literature mean value (7.92) reported by [29] studied in Ethiopia and also similar to another literature mean value (12 ± 0.3) reported by [30] in Ethiopia. The total mean fat content of back cumin (32.13%) was greater than the fat content of fenugreek (12.8%) cultivated in the Gamo zone; at the 0.05 level, the means were significantly different.

#### 3.1.5. The Crude Protein Content

Protein is essential for normal growth, body development, and general repair of body tissues, enzymes, and hormones as well as other substances required for healthy functioning. As shown in Table 2, the average crude protein content of the three cultivars of black cumin (Kamba, Daramalo, and Dita) was 19.8 ± 0.14%, 21.8 ± 0.23%, and 21.2 ± 0.26%, respectively. These showed that black cumin from Daramalo has the highest (21.774%) and black cumin from Kamba has the lowest (19.8 ± 0.14%) crude protein content. At the 0.05 level, the means are not significantly different (*p* = 0.972, which is *p* > 0.05). The total average protein content of black cumin (20.9 ± 0.09%) was smaller than the average protein content of fenugreek (30.7 ± 0.09%), and at the 0.05 level, the means are significantly different. The average crude protein content of three cultivars of black cumin (20.9 ± 0.09) is higher than the literature value (18.9 ± 0.82) reported by [30], smaller than the literature mean value (28.0 ± 0.36) reported by [28], and in agreement with the value (20.3%) studied by [27]. The average crude protein content of fenugreek samples collected from Kamba, Daramalo, and Dita woredas was 30.6 ± 0.11%, 29.7 ± 0.25%, and 32.0 ± 0.38%, respectively. The result of the study showed that fenugreek from Dita has the highest crude protein content (32.0%) and fenugreek from Daramalo has the lowest crude protein content. At 0.05 levels, the means are not significantly different. The total average crude protein content in fenugreek (30.8 ± 0.05%) of this study was greater than literature mean values of 29.31% and 19.8 ± 0.3% presented in [29, 30], respectively. This was because of the differences in climate, geographical, and mineral conditions of the cultivation area.

#### 3.1.6. Total Carbohydrate Content

Carbohydrate is the main source of energy in the human body. The average carbohydrate content of black cumin samples collected from Kamba, Daramalo, and Dita woredas was 34.4 ± 0.93%, 33.6 ± 0.95%, and 34.3 ± 0.35%, respectively. The highest carbohydrate content was observed in black cumin from Kamba, and black cumin from Daramalo has the lowest carbohydrate content. At the 95% confidence level, the means were not significantly different (*p* = 0.22036, which is *p* > 0.05). The overall carbohydrate mean value of 34.1 ± 0.73% in black cumin in this study was greater than literature mean value (19.7% and 30) [27, 28]. The carbohydrate content of black cumin was significantly smaller than that of fenugreek. The average carbohydrate content in fenugreek samples collected from the Kamba, Daramalo, and Dita woredas was 46.9 ± 0.75%, 46.1 ± 0.54%, and 45.1 ± 0.56%, respectively. Fenugreek from Kamba has the highest carbohydrate content (46.9 ± 0.75), and that from Dita has the lowest carbohydrate content (45.1 ± 0.56%). At the 95% confidence level, the means were not significantly different (*p* = 0.91805, which is *p* > 0.05). The overall mean carbohydrate content in three cultivars of fenugreek (46.0 ± 0.51%) was greater than the literature mean value (45.21% and 45) studied in Ethiopia [29, 30] and significantly smaller than the literature value [33].

#### 3.1.7. Gross Energy Value

As shown in Table 2, the gross energy content of black cumin collected from Kamba, Daramalo, and Dita was 498 ± 4.4 kcal/100 g, 521 ± 6.4 kcal/100 g, and 507 ± 4.3 kcal/100 g, respectively, and their average energy was 509 ± 3.3 kcal/100 g. Black cumin from Daramalo has the highest energy, and that from Kamba has the lowest energy value. At the 0.05 level, the means are not significantly different. The gross energy values of fenugreek samples from Kamba, Daramalo, and Dita were 430 ± 5.8 kcal/100 g, 4062 ± 1.4 kcal/100 g, and 430 ± 1.5 kcal/100 g, respectively, with the total average energy content of three cultivars of 422 ± 1.6 kcal/100 g. At the 0.05 level, the two means are not significantly different (*p* = 0.96548, which was *p* > 0.05). The gross energy content of black cumin was significantly greater than the gross energy content of fenugreek.

### 3.2. Determination of Phytochemical

#### 3.2.1. Quantitative Determination of Phytochemicals

The data are provided in Table 3 as mean ± SD. The quantitative measurement of a few chosen phytochemicals (total phenolics, total flavonoid, and total alkaloid content) in black cumin and fenugreek was made using a UV-Vis spectrometer using a different standard solution calibration curve. Since percentage relative standard deviation (% RSD) values for all quantification data were in the range of 4.506 to 9.081, which is less than 10, this shows good precision of the measurement data.

#### 3.2.2. Total Phenolic Content in Black Cumin and Fenugreek

The results indicated that black cumin from Dita has the highest and that from Kamba has the lowest phenolic content with total average phenolic content of 193 ± 5.3. At 0.05 levels, there was no significant difference between the means of the total phenolic content in three cultivars of black cumin. The TPC in black cumin of this study was greater than the literature mean value of160 ± 11 mg DAE/100 g [35] and less than the literature mean values of 480 mg GAE/100 g and 589 ± 0.02 mg GAE/100 g [36]. The total phenolic content in fenugreek from Daramalo was highest and that from Kamba was lowest, but at 0.05 levels, there was no significant difference between the means of three cultivars of fenugreek. The total average phenolic content in this study was higher than the literature mean value of 139.2 mg GAE/100 g reported in [37] and significantly lower than the literature mean values of589 ± 0.02 mg GAE/100 greported in [38]. This was because of climatic, geographical, and soil type differences. The phenolic content in fenugreek was higher than that in black cumin. This was because of variation in plant variety.

#### 3.2.3. Total Flavonoid Content in Black Cumin and Fenugreek

Flavonoids have been reported to exert a wide range of biological activities such as anti-inflammatory, antibacterial, antiallergic, cytotoxic antitumor, treatment of neurodegenerative diseases, vasodilator activities, and inhibition of lipid peroxidation [39]. The total flavonoid content in black cumin was lower than that in fenugreek. The total flavonoid content of black cumin cultivated in Dita was highest and that in Kamba was lowest. At the 0.05 level, the means were not significantly different (*p* = 0.98765, which was *p* > 0.05). The total flavonoid content in black cumin of this study was higher than the literature mean values of 14.0 ± 0.8 reported by [39] and smaller than the literature mean value of 3.78 mg reported by [39]. The flavonoid content in fenugreek from Daramalo was highest and from Kamba was lowest, but at the 0.05 level, the means were not significantly different (*p* = 0.18629, which was *p* > 0.05). The total average flavonoid content in fenugreek of this study was lower than the literature mean values (145 mg QE/100 g and 274 mg QE/100 g) reported by [40]. In general, the result of this study showed that black cumin and fenugreek cultivated in the Gamo zone were good sources of flavonoid compounds.

#### 3.2.4. Total Alkaloid Content of Black Cumin and Fenugreek

Alkaloids protect against chronic diseases (reducing headaches associated with hypertension). Alkaloids are a diverse group of secondary metabolites found to have antimicrobial activity by inhibiting DNA topoisomerase [23]. The total alkaloid content in black cumin from Daramalo was highest and that from Kamba was lowest. At the 0.05 level, the means are not significantly different (*p* = 0.99156, which was *p* > 0.05). The total alkaloid content in black cumin was higher than the alkaloid content in fenugreek, and at the 95% confidence level, the two means were significantly different. This was because of sample variate difference. The alkaloid content in fenugreek from Dita was highest and that from Daramalo was lowest, but at 0.05, there was no significant difference between means.

### 3.3. Determination of Heavy Metals Using AAS

#### 3.3.1. Calibration of Instrument

The intermediate standard solution (100 mg/L) was diluted with distilled water to create five calibration standard solutions for each heavy metal. These solutions were then tested using the same process as samples, and their absorbance readouts were recorded from FAAS. The linear regression equations (calibration curves) were derived from the standard solution concentration and its absorbance readout. It was determined that the linearity of the established calibration curves is good, and the results are correct, because the correlation coefficients (*R*^2^) of all the calibration curves were >0.995, which demonstrated that there was good correlation (connection) between concentration versus absorbance.

#### 3.3.2. Method Validation

Validation is one of the most important measures that analytical chemists use to predict whether a method meets the needs of intended purpose or not. Accuracy (recovery test), precision, and method detection limit were used to check the validity of the instrument, sample preparation, and measurement methods used through this research.

*(1) Precision*. The precision is the measure of closeness of the results obtained from the triplicate analysis of the same sample under the same condition. It is usually expressed as variance, standard deviation, or percent relative standard deviation of a set of measurements. For this research, the precision of the results was evaluated by the standard deviation and percent relative standard deviation of the results of the triplicate analysis of the study samples. As indicated in Table 4, the standard deviations (SD) of the determined heavy metals in the study samples were ranges between 0.0682 and 5.7231 and percentage relative standard deviations (% RSD) were ranges from 0.1858 to 9.88% which mean less than 10% for all the analyzed elements. This indicates that there is good precision (agreement) between the measurements.

*(2) Recovery Test*. The reliability and efficiency of the procedure were checked by spiking a known concentration of heavy metals to the sample. As indicated in Table 5, the percent recovery obtained lies in the range from 90.75 to 102.56 which was within acceptable ranges (100 ± 15). These values indicate that the analytical procedure used in this research was appropriate and valid for the analysis of selected metals in study samples.

#### 3.3.3. The Level of Heavy Metals in Black Cumin and Fenugreek

The concentrations of trace heavy metals (Fe, Zn, Cu, Mn, Co, Cr, and Ni) and toxic heavy metals (Pb and Cd) in black cumin and fenugreek collected from three different woredas of the Gamo zone were determined by using FAAS. The mean concentrations of these heavy metals in the study samples were determined from their triplicate analysis. The mean concentrations of metals in mg/kg by dry weight basis with their respective SD are reported in Table 4. Thus, significant difference of concentration of each metal among the study samples was checked by comparing the *p* values at the 95% confidence level. If *p* ≥ 0.05 at the 95% confidence level, the means are not significantly different, and if *p* < 0.05, the means are significantly different.

As indicated in Table 4, the concentrations of selected heavy metals were successfully determined in study samples except Cd and Pb which were not detected. The total average mean concentration of determination of heavy metals in three cultivars of study samples represents the Gamo zone study area result.

*(1) Iron*. Iron has several key functions in the human body as it is a constituent of certain biological molecules like hemoglobin and is involved in various physiological activities including oxygen supply, energy production, and immunity [41]. However, iron overdose is associated with an adverse effect on various metabolic functions and the cardiovascular system [42]. Iron concentrations in black cumin and fenugreek samples collected from three woredas in the Gamo zone varied between 74.934 ± 2.425 and 172.632 ± 0.321 mg/kg. These values were relatively higher than the concentration values of other heavy metals. Iron concentrations in black cumin from Kamba, Daramalo, and Dita were 85.3 ± 1.6, 75.2 ± 5.7, and 74.98 ± 2.4, respectively, with a total average iron concentration of 78.5 ± 5.9. At the 0.05 level, the means were not significantly different (*p* = 0.904, which was *p* > 0.05). The iron concentration in black cumin (78.4612 ± 5.8819 mg/kg) of this study is higher than the literature mean value (59.3 mg/kg) reported by [43] and is significantly lower than the literature mean value (150 ± 1.0 mg/kg) reported by [43] studied in Bangladesh. Iron concentrations in fenugreek from Kamba, Daramalo, and Dita were 170 ± 3.3 mg/kg, 162 ± 1.7 mg/kg, and 172 ± 0.32 mg/kg, respectively, with a total mean of three cultivars of 168 ± 5.2 mg/kg. At the 0.05 level, the means were not significantly different (*p* = 0.8489, which was *p* > 0.05). Iron content in black cumin was significantly smaller than that in fenugreek; at the 0.05 level, the means are significantly different (*p* = 1.54103*E*^−4^; *p* < 0.05). The mean value iron concentration in three cultivars of fenugreek of this study (168 ± 5.2 mg/kg) was higher than the literature mean value of 72.7 mg/kg studied in Ethiopia [44] and significantly smaller than the literature mean value of 540 ± 2.6 mg/kg [45]. The results of the iron concentration in both black cumin and fenugreek were below the tolerable daily intake (TDI) value (0.8 mg/kg bw per day) regulated by EFSA and FAO/WHO (2010).

*(2) Zinc (Zn)*. It is known that zinc is an essential trace element not only for humans but for all organisms. It is a component of over 300 enzymes and an even greater number of other proteins, which emphasizes its indispensable role for human health [41]. Zinc concentration in black cumin and fenugreek was found to be in the range between 13.2 ± 0.24 mg/kg and 28.7 ± 0.18 mg/kg. Zinc concentrations in black cumin collected from Kamba, Daramalo, and Dita were 25.8 ± 0.13 mg/kg, 27.3 ± 1.07 mg/kg, and 28.7 ± 0.18 mg/kg with a total value of 27.3 ± 0.34 mg/kg. Zinc concentration in black cumin from Dita was highest and that from Kamba was lowest. At the 0.05 level, the means were not significantly different (*p* = 0.77714, which was *p* > 0.05). The total mean of zinc concentration in black cumin of this study was smaller than the literature mean value (47.0 ± 2.1 mg/kg) [46] and smaller than the literature mean value reported by [37]. The result of the zinc concentration in black cumin of the current work was similar to that in previous works. Zinc concentrations in fenugreek samples collected from Kamba, Daramalo, and Dita were 13.2268 ± 0.2389 mg/kg, 15.9 ± 0.22 mg/kg, and 15.1 ± 0.28 mg/kg, respectively; the total mean concentration of three cultivars was 14.8 ± 0.20 mg/kg. At the 0.05 level, the two means are not significantly different (*p* = 0.44621, which was *p* > 0.05). Zinc concentration in fenugreek of this study was higher than the literature mean value (6.1 ± 0.1 mg/kg) reported by [45], and it was significantly smaller than the literature value (45 mg/kg) result reported by [44]. The concentration of zinc in fenugreek was slightly greater than in black cumin; at the 0.05 level, the means are significantly different (*p* = 4.26778*E*^−4^; *p* < 0.05); in both samples, the concentration was below the tolerable daily intake value (TDIV) which is 0.43 mg/kg bw per day.

*(3) Copper (Cu)*. The level of copper in black cumin and fenugreek samples ranges in between 2.77 ± 0.16 mg/kg and 5.76 ± 0.24 mg/kg. Copper concentrations in black cumin collected from Kamba, Daramalo, and Dita were 2.76 ± 0.16 mg/kg, 3.13 ± 0.20 mg/kg, and 3.27 ± 0.28 mg/kg, respectively, with a total mean concentration of 3.06 ± 0.26 mg/kg. The black cumin from Dita has the highest copper content and that from Kamba has the lowest copper content. At the 0.05 level, the means are not significantly different (*p* = 0.74467, which was *p* > 0.05). Total mean concentration of copper in black cumin (3.06 ± 0.26) of the current work was compared to different literature values; it was smaller than the literature mean value (13.7 ± 0.24 mg/kg) reported by [46], and it was also lower than values (13.80 mg/kg) reported by [43] and slightly higher than the literature mean value (2.34) reported by [37]. Copper concentrations (mg/kg) in fenugreek collected from Kamba, Daramalo, and Dita were 5.76 ± 0.24, 3.69 ± 0.24, and 4.83 ± 0.20, respectively, with a total average of 4.76 ± 0.07. At the 0.05 level, there was no significant difference between means (*p* = 0.70393, which was *p* > 0.05). Copper content in black cumin was smaller than that in fenugreek; at the 0.05 level, the two means were not significantly different (*p* = 0.05059; *p* > 0.05). The total mean concentration (4.76 ± 0.07) of the current finding was smaller than the literature mean values of 11.6 ± 1.5[44] and 8.25 [45]. It was higher than other study values reported by [47].

*(4) Manganese (Mn)*. Concentrations of manganese in black cumin collected from Kamba, Daramalo, and Dita were 7.93 ± 0.27 mg/kg, 8.54 ± 0.18 mg/kg, and 8.94 ± 0.17 mg/kg, respectively, and their total mean concentration was 8.47 ± 0.21. At the 0.05 level, the two means are not significantly different (*p* = 0.25273, which was *p* > 0.05). The total mean concentration (mg/kg) of manganese in black cumin of the current result (8.46 ± 0.21) was smaller than the literature mean value (45.0 ± 1.2 mg/kg) studied by [46] and higher than the other result reported by [43]. The results of this study finding are similar with other previous studies [37] with slight variations. Concentrations (mg/kg) of manganese in fenugreek samples from Kamba, Daramalo, and Dita were 14.1 ± 0.21 mg/kg, 13.3234 ± 0.2467 mg/kg, and 13.7 ± 0.24 mg/kg, respectively, and the total mean value was 13.72 ± 0.40 mg/kg. At the 0.05 level, the two means are not significantly different (*p* = 0.08995; *p* > 0.05). The concentration of manganese in fenugreek was significantly greater than that in black cumin; at the 0.05 level, the two means were significantly different (*p* = 1.48947*E*^−4^, which was *p* < 0.05). Manganese concentration in fenugreek of this study was smaller than the results in previous studies (73.0 ± 1.34) reported by [45] and higher than the other literature value reported by [47].

*(5) Cobalt (Co)*. The cobalt concentration ranges from 3.36 ± 0.24 to 10.4 ± 1.03 mg/kg. The cobalt content in black cumin from Kamba, Daramalo, and Dita was 9.43 ± 0.16 mg/kg, 10.3 ± 0.18 mg/kg, and 10.4 ± 1.0 mg/kg, respectively, with a total mean value of 10.0 ± 0.37 mg/kg. At the 0.05 level, the two means are not significantly different (*p* = 0.92586, which was *p* > 0.05). Cobalt concentrations in fenugreek from Kamba, Daramalo, and Dita were 3.74 ± 0.22 mg/kg, 3.83 ± 0.10 mg/kg, and 3.36 ± 0.24 mg/kg, respectively; their total mean concentration was 3.656 ± 0.25 mg/kg. The means were not significantly different at the 0.05 level (*p* = 0.78222; *p* > 0.05). Cobalt in the fenugreek sample was significantly greater than that in black cumin; at the 0.05 level, the means are significantly different (*p* = 4.67376*E*^−5^, which was *p* < 0.05). Cobalt content obtained in black cumin was slightly greater than the literature mean value (8.5 ± 0.1) reported by [37], and it was smaller than another study result (15.3 ± 1.2) [46]. Cobalt content in both black cumin and fenugreek samples was below the standards of tolerable daily intake value (0.023) regulated by the FSA (2003).

*(6) Nickel (Ni)*. Nickel was detected only in fenugreek samples, not in black cumin. Nickel content in fenugreek samples from Kamba, Daramalo, and Dita was 1.745 ± 0.10 mg/kg, 2.40 ± 0.07 mg/kg, and 2.22 ± 0.17 mg/kg, respectively, with a total average of 2.12 ± 0.03 mg/kg. At the 0.05 level, the two means are not significantly different (*p* = 0.59255; *p* > 0.05). The average nickel concentration in fenugreek was higher than the literature mean value (0.340 mg/kg) [44]. Nickel content was below tolerable daily intake value (0.0028 mg/kg bw per day).

*(7) Chromium (Cr), Lead (Pb), and Cadmium (Cd)*. Chromium (Cr), lead (Pb), and cadmium (Cd) were not detected in both black cumin and fenugreek samples. Thus, black cumin and fenugreek cultivated in the Gamo zone were free from the above metal toxicity.

## 4. Conclusion

This research looked at the phytochemicals, heavy metals, and proximate composition of fenugreek and black cumin grown in the Gamo region. The investigation's findings showed that fenugreek samples had higher fiber, protein, and carbohydrate content than black cumin samples did in terms of moisture, ash, fat, and energy values. We measured the amounts of various significant phytochemicals (phenolics, flavonoids, and alkaloids), and the results showed that fenugreek had higher concentrations of phenolics and flavonoids, and black cumin had higher concentrations of alkaloids than fenugreek. The quantitative findings of the identified phytochemicals agreed with earlier research. Fe, Zn, Cu, Mn, Co, Cr, Ni, Pb, and Cd were found in both black cumin and fenugreek, but Ni was only found in fenugreek, and Cr, Pb, and Cd were not found in either sample, according to the analysis of heavy metals (Fe, Zn, Cu, Mn, Co, Cr, Ni, Pb, and Cd). The results were consistent with other research, and the concentrations of all determined heavy metals were below the limits of tolerable daily intake values. The concentrations of all detected heavy metals were in the range of nutritionally adequate levels. The nutritional composition of black cumin and fenugreek seed was found to be in close conformance with values already described in the literature with a few minor deviations, according to the study's findings. This little variation might be the result of several elements that vary depending on the type of seed purchased from the location. The temperature, geography, soil, and other variables may be to blame for these changes.

## Figures and Tables

**Table 1:** Series of working standard solutions of metals.

S. no.	Metals	Series (in *μ*L)
1	Fe	50, 150, 250, 350, and 450
2	Zn	50, 150 L, 250, 350, and 450
3	Cr	50, 150, 250, 350, and 450
4	Cu	125, 250, 375, 500, and 625
5	Ni	125, 250, 375, 500, and 625
6	Mn	50, 100, 150, 200, and 250
7	Co	25, 125, 250, 375, and 500
8	Cd	25, 125, 250, 375 L, and 500
9	Pb	5, 10, 15, 20, and 25

**Table 2:** The proximate composition of three cultivars of Bc and Fg cultivated in the Gamo zone.

Parameters	Bc-K	Bc-Dr	Bc-Dt	Fg-K	Fg-Dr	Fg-Dt
Moisture (%)	6.48 ± 0.16	6.69 ± 0.06	6.92 ± 0.04	6.08 ± 0.05	5.90 ± 0.07	5.99 ± 0.15
Ash (%)	5.23 ± 0.04	5.05 ± 0.02	4.79 ± 0.13	4.27 ± 0.04	4.44 ± 0.06	4.10 ± 0.14
Fiber (%)	6.84 ± 0.28	6.80 ± 0.29	7.07 ± 0.08	9.48 ± 0.30	9.54 ± 0.34	9.16 ± 0.36
Fat (%)	32.1 ± 0.53	33.5 ± 0.24	30.7 ± 0.18	13.3 ± 0.91	11.5 ± 0.22	13.5 ± 0.27
Protein (%)	19.8 ± 0.13	21.8 ± 0.23	21.29 ± 0.26	30.6 ± 0.11	29.7 ± 0.25	32.0 ± 0.37
Carbohydrate (%)	34.4 ± 0.93	33.6 ± 0.95	34.4 ± 0.34	46.9 ± 0.75	46.1 ± 0.50	45.1 ± 0.56
Energy (kcal/100 g)	498 ± 4.4	521 ± 6.5	507 ± 4.3	430 ± 5.8	406 ± 1.4	430 ± 1.6

**Table 3:** Content of selected phytochemicals (mean ± SD).

Phytochemicals	Bc-K	Bc-Dr	Bc-Dt	Fg-K	Fg-Dr	Fg-Dt
TPC (mg GAE/100 g)	179 ± 11	182 ± 12	218 ± 10	326 ± 29	358 ± 18	461 ± 29
TFC (mg QE/100 g)	83.2 ± 7.0	88.7 ± 8.2	90.9 ± 4.6	108 ± 8.9	120 ± 2.8	120 ± 6.5
TAC (mg AE/100)	62.2 ± 3.4	73.7 ± 5.2	72.5 ± 6.9	37.9 ± 9.1	30.0 ± 2.8	44.9 ± 3.5

TPC = total phenolics content; TFC = total flavonoid content; TAC = total alkaloid content.

**Table 4:** Mean concentrations (mg/kg) of metals in black cumin and fenugreek.

Samples	Fe	Zn	Cu	Mn	Co	Cr	Ni	Pb	Cd
Fg-K	170 ± 3.3	13.2 ± 0.24	5.76 ± 0.24	14.1 ± 0.21	3.74 ± 0.22	ND	1.74 ± 0.10	ND	ND
Fg-Dr	162 ± 1.68	15.9 ± 0.22	3.62 ± 0.24	13.3 ± 0.25	3.83 ± 0.10	ND	2.40 ± 0.07	ND	ND
Fg-Dt	172 ± 0.32	15.1 ± 0.27	4.83 ± 0.20	13.7 ± 0.23	3.36 ± 0.24	ND	2.22 ± 0.17	ND	ND
Bc-K	85.3 ± 1.6	25.8 ± 0.13	2.76 ± 0.16	7.93 ± 0.28	9.44 ± 0.16	ND	ND	ND	ND
Bc-Dr	75.2 ± 5.72	27.3 ± 1.0	3.14 ± 0.20	8.55 ± 0.18	10.3 ± 0.18	ND	ND	ND	ND
Bc-Dt	74.9 ± 2.42	28.6 ± 0.18	3.26 ± 0.28	8.94 ± 0.17	10.4 ± 1.03	ND	ND	ND	ND

Key: ND: not detected.

**Table 5:** Recovery test result of the heavy metal determination.

Elements		Fg-K	Fg-Dr	Fg-Dt	Bc-K	Bc-Dr	Bc-Dt
Fe	% recovery	98.6 ± 5.5	97.1 ± 3.2	97.6 ± 2.7	105 ± 0.86	101 ± 5.6	100 ± 6.5
Zn	% recovery	100 ± 2.8	95.0 ± 0.69	104 ± 3.9	102 ± 1.5	99.3 ± 5.6	106 ± 2.9
Cu	% recovery	95 ± 1.2	102 ± 0.82	96.5 ± 0.81	93.4 ± 2.4	94.2 ± 1.5	92.2 ± 1.1
Mn	% recovery	99.6 ± 2.2	95.7 ± 1.8	93.8 ± 1.9	100 ± 1.3	95.8 ± 1.9	99.7 ± 2.5
Co	% recovery	99.5 ± 2.2	95.7 ± 1.8	93.8 ± 1.9	100 ± 1.2	95.79 ± 1.9	99.7 ± 2.5
Cr	% recovery	—	—	—	—	—	—
Ni	% recovery	101 ± 2.5	98.7 ± 0.71	93.5 ± 1.7	—		—
Pb	% recovery	—	—	—	—	—	—
Cd	% recovery	—	—	—	—	—	—

## Data Availability

Data will be available upon request.

## Abbreviations

Bc: Black cumin
Fg: Fenugreek
K: Kamba
Dr: Daramalo
Dt: Dita.
